# STIFLED UTILIZATION OF DEMENTIA-RELATED HEALTHCARE SERVICES DUE TO STIGMA IN RURAL APPALACHIA

**DOI:** 10.1093/geroni/igac059.188

**Published:** 2022-12-20

**Authors:** Elizabeth Rhodus, Allison Gibson, David Gross, Rob Sprang, Kelly Parsons, Julia Johnston, Gregory Jicha

**Affiliations:** University of Kentucky, Lexington, Kentucky, United States; University of Kentucky, Lexington, Kentucky, United States; St. Claire Healthcare, Morehead, Kentucky, United States; University of Kentucky, Lexington, Kentucky, United States; University of Kentucky, Lexington, Kentucky, United States; University of Kentucky, Lexington, Kentucky, United States; University of Kentucky, Lexington, Kentucky, United States

## Abstract

Residency in rural Appalachia is linked with heightened morbidity and mortality due to a myriad of conditions, many of which are associated with increased risk and prevalence of Alzheimer’s disease and related dementias (ADRD). Despite this, access to and utilization of dementia-specific healthcare services in the region are limited. This study presents community-based stigma associated with enrollment in healthcare clinical research offered in rural Appalachia. Additional data from focus groups with care partners of people with memory impairment in rural Appalachia discuss implications of stigma in their communities. Findings elaborate on recruitment challenges associated with terminology, such as caregiver and dementia, as well as availability of diagnosticians. This study illustrates unique characteristics needed for community-based education programs tailored to the culture and customs of rural regions in order to increase utilization of healthcare for older adults at risk or living with ADRD.

